# Applicability of the WHO maternal near miss tool in sub-Saharan Africa: a systematic review

**DOI:** 10.1186/s12884-019-2225-7

**Published:** 2019-02-26

**Authors:** Abera Kenay Tura, To Lam Trang, Thomas van den Akker, Jos van Roosmalen, Sicco Scherjon, Joost Zwart, Jelle Stekelenburg

**Affiliations:** 1 School of Nursing and Midwifery, College of Health and Medical Sciences, Haramaya University, Dire Dawa, Ethiopia; 2 Department of Obstetrics and Gynecology, University Medical Centre Groningen, University of Groningen, Hanzeplein 1, 9700 RB, P.O.B, 30 001 Groningen, The Netherlands; 3 Department of Obstetrics and Gynecology, Leeuwarden Medical Center, Leeuwarden, The Netherlands; 4 Department of Obstetrics and Gynaecology, Leiden University Medical Center, Leiden, the Netherlands; 5 Athena Institute, Vrije Universiteit Amsterdam, Amsterdam, The Netherlands; 6 Department of Obstetrics and Gynecology, Deventer Ziekenhuis, Deventer, The Netherlands; 7 Department of Health Sciences, Global Health, University Medical Centre Groningen, University of Groningen, Groningen, The Netherlands

**Keywords:** Systematic review, Severe acute maternal morbidity, Maternal near miss, Severe maternal outcomes, Sub-Saharan Africa

## Abstract

**Background:**

Applicability of the World Health Organization (WHO) maternal near miss criteria in low-income settings is not systematically addressed in the literature. The objective of this review was to determine the applicability of the WHO maternal near miss tool in sub-Saharan Africa.

**Methods:**

We searched PubMed, Embase, Popline, CINAHL, AJOL, and Google scholar using key words for maternal near miss and sub-Saharan Africa. Studies which applied the WHO maternal near miss criteria, containing clear definitions, and published between January 1st, 2009 and December 31st, 2017 were included. Two authors independently extracted data. Quantitative analysis and narrative synthesis were conducted, and medians with interquartile range (IQR) were calculated for summarizing the findings. Methodological quality of the studies was assessed using the Estabrook’s quality assessment and validity tool.

**Results:**

Fifteen studies from nine countries comprising 227,077 participants were included. Median maternal near miss ratio was 24.2 (IQR: 12.4–35.8) per 1000 live births ranging from 4.4 in a population-based study in South Africa to 198 in a rural private hospital in Nigeria. Eight studies reported challenges in implementing the WHO maternal near miss tool, especially related to the threshold for blood transfusion, and availability of several laboratory-based criteria. In three studies, local adaptations were made.

**Conclusion:**

This review showed that the WHO maternal near miss tool is not uniformly applied in sub-Saharan Africa. Therefore, a common adaptation for the region is required to increase its applicability.

**Electronic supplementary material:**

The online version of this article (10.1186/s12884-019-2225-7) contains supplementary material, which is available to authorized users.

## Background

With the decline of maternal deaths, studying maternal near misses (MNM) has been used as a proxy to measure quality of obstetric care [1, 2]. MNM refers to a very ill pregnant or delivered woman who nearly died but survived a complication during pregnancy, childbirth or within 42 days of termination of pregnancy [3]. Studying MNM has additional advantages to studying maternal deaths since it occurs more often, shares similar characteristics with deaths and is less ‘threatening’ to report by health providers and managers, possibly reducing underreporting [1, 4, 5]. In addition, audit of MNM brings the possibility to include opinions and perceptions of the women themselves, who may be interviewed after the event [6, 7].

A WHO maternal morbidity-working group developed MNM criteria in 2009 mainly focusing on presence of organ dysfunction [3]. The WHO near-miss approach was published in 2011 to serve as a manual for conducting MNM studies [6]. The manual provides guidelines to implement MNM studies (including definition of terms and expected results), calculations of MNM indicators, a data collection tool, and dummy tables, as well as guidance for interpretation. The MNM identification criteria consist of 25 parameters grouped into clinical, laboratory, and management-based criteria mainly focusing on presence of organ dysfunction—cardiac, respiratory, renal, coagulation/ hematological, hepatic, neurologic, and uterine dysfunctions (Table 1). Although the WHO MNM tool has been widely used, including in low-income settings, the tool turned out to be rather difficult to apply because of limited applicability especially the laboratory- and management-based criteria in low-income settings [8–10]. Therefore, several authors suggested local adaptations [9, 11], noted the need for practical MNM criteria for use in low-resource settings [8].

**Table 1:** World health organization maternal near miss criteria [3]

Clinical criteria	
Acute cyanosis	Loss of consciousness lasting > 12 h
Gasping	Loss of consciousness and absence of pulse/heart beat
Respiratory rate > 40 or < 6/min	Stroke
Shock	Uncontrollable fit/total paralysis
Oliguria non-responsive to fluids or diuretics	Jaundice in the presence of pre-eclampsia
Clotting failure	
Laboratory-based criteria	
Oxygen saturation < 90% for > 60 min	pH < 7.1
PaO2/FiO2 < 200 mmHg	Lactate > 5
Creatinine > 300 mmol/l or > 3.5 mg/dl	Acute thrombocytopenia (< 50,000 platelets)
Bilirubin > 100 mmol/l or > 6.0 mg/dl	Loss of consciousness and the presence of glucose and ketoacids in urine
Management-based criteria	
Use of continuous vasoactive drugs	Intubation and ventilation for > 60 min not related to anesthesia
Hysterectomy following infection or hemorrhage	Dialysis for acute renal failure
Transfusion of u5 units red cell transfusion	Cardio-pulmonary resuscitation (CPR)

Systematic reviews have indicated that the use of different sets of criteria was one of the major limitations in estimating the burden of MNM, hampering comparisons between settings and countries [12–14]. Despite WHO’s recommendation to use a uniform set of clinical, laboratory-, and management- based criteria for MNM identification [3], classifications based on only disease-based criteria are still being applied in several studies [15]. Any recommendations to apply either the WHO MNM criteria or resorting to adaptations for low-resource settings should be based on knowledge of performance of available criteria and pay attention to challenges that may occur during their implementation. Aim of this review was to assess applicability and challenges related to use of the WHO MNM tool in sub-Saharan Africa.

## Methods

The review was conducted according to the Preferred Reporting Items for Systematic reviews and Meta-Analysis (PRISMA) guideline [16]. The review protocol was registered in PROSPERO (CRD42015023883). PubMed, Embase, Popline, CINHAL, and AJOL databases were searched using key terms developed in consultation with a medical information specialist librarian of the University Medical Centre Groningen. We used the key terms ‘near miss’, ‘severe acute maternal morbidity’, ‘severe maternal morbidity’ in combination with terms used to describe sub-Saharan African region (Additional file 1). Open grey sources and references of identified articles were also searched for additional publications. The search was updated on December 28, 2018.

All identified articles were exported to Refworks reference manager and duplicates removed. Two reviewers (AKT and TLT) independently screened titles and abstracts of the studies. All potentially relevant articles and articles that could not clearly be excluded on the basis of the abstract only were retained for full text review. Differences between assessors to include articles in full text review were resolved by a senior reviewer (JS). Abstract and full text screening were conducted online using Covidence (www.covidence.org) [17]. Studies were included in the review if conducted in sub-Saharan Africa; provided a clear definition of MNM and used the WHO MNM criteria (or adaptations); were published between January 1, 2009 and December 31, 2017; used defined denominators (live births or deliveries); and contained data on frequency of MNM. Also included were studies that contained qualitative data of relevance to assessing the use of the tool, in line with the objective of this review. Studies that did not apply the WHO MNM criteria or that provided no primary data i.e. conference abstracts, reviews, and case reports were excluded. Qualitative studies were excluded since their main focus is mainly on description of the MNM experience: quality of life, risk of complications after MNM, social or economic impacts or experience of women regarding their treatment or complications [18–24]. The year 2009 was chosen as the initial year of inclusion, since this was the year of publication of the 2009 WHO MNM tool and 2017 was the most recent year at the time our search was conducted. AKT and TLT collected data on study design, study settings, data collection period, denominators, number of participants, MNM, maternal deaths, and qualitative data related to applicability or adaptation of the criteria. Data were extracted online using a systematic review data repository (srdr.ahrq.gov) platform [25]. Conflicts during data collection were resolved by discussion until unanimity was reached.

One author (AKT) assessed the methodological quality of all studies using Estabrook’s quality assessment and validity tool for cross sectional studies [26, 27]. Estabrook’s quality assessment and validity tool, developed based on the Cochrane collaboration guidelines, has been widely used for assessing methodological quality of cross-sectional studies [26, 27]. The tool contains a maximum of 16 points and comprises three core areas: sampling, measurement, and statistical analysis. Each item contains a one-point score (0 or 1) except two items (representativeness and matching) containing scores from 0 to 2. A final score for each study was derived using the scoring system developed by de Vet et al [28] by dividing the total score obtained by total points possible after subtracting total number of not applicable (16-not applicable), resulting in a final score between 0 and 1. Each study was then classified as weak (< 0.5), moderately-weak (0.51–0.65), moderately-strong (0.66–0.79), or strong (> 0.80).

Reported challenges related to the use of the WHO MNM tool and qualitative remarks about the applicability of the tool were synthesized using texts and tables. Medians with interquartile range were used to present MNM ratio, maternal mortality ratio (MMR) and mortality index (MI). We calculated MNM ratio (MNM cases per 1000 live births), MMR (maternal deaths per 100,000 live births) and mortality index (maternal deaths divided by the sum of maternal deaths and MNM). These MNM indicators are essential components of MNM studies and give an indication of the performance of the MNM tool and the quality of care in a particular context [6].

## Results

### General description of studies

**Fig. 1:**
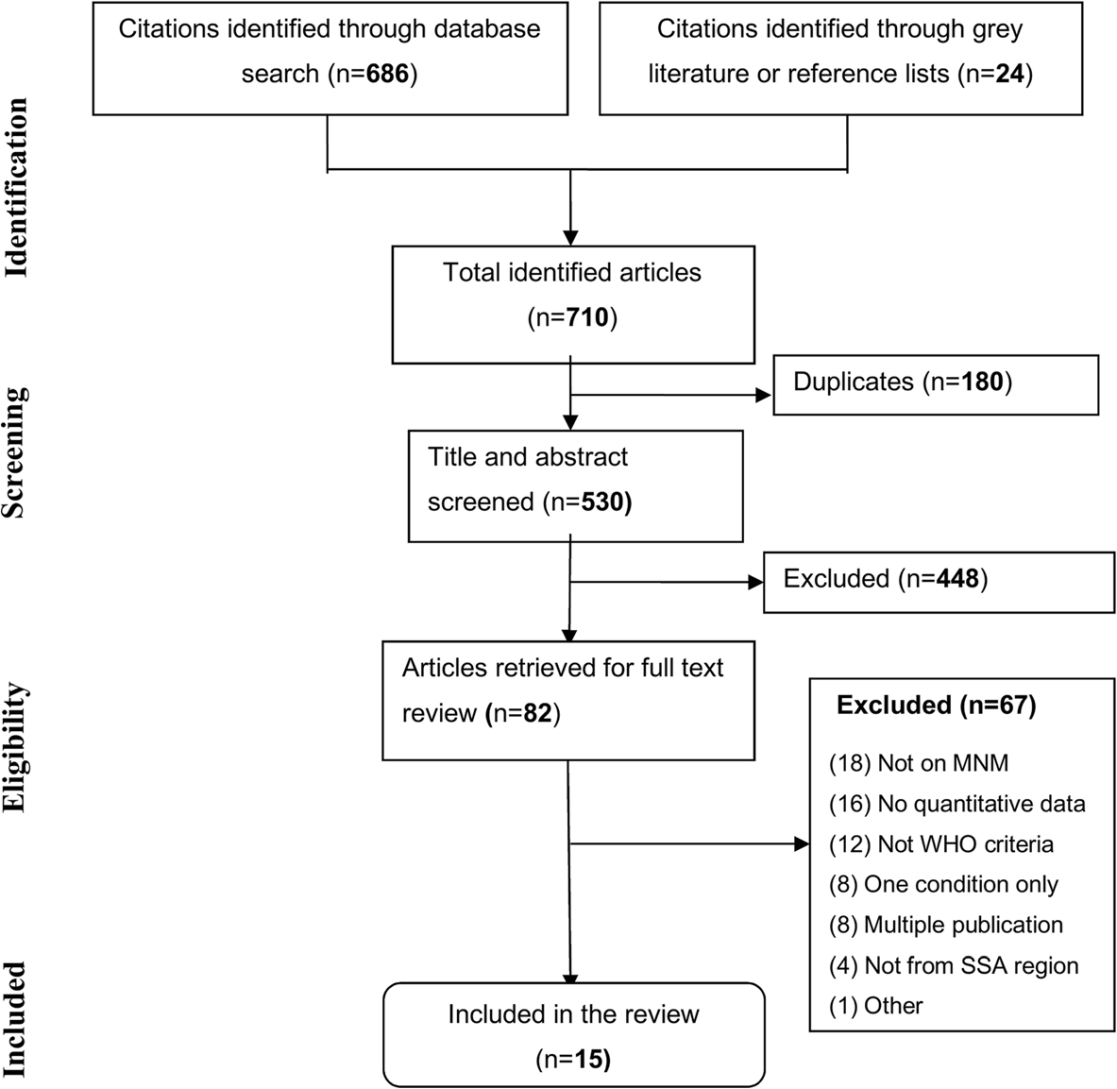
PRISMA flow chart of the overall phases of the systematic review [16]

A total of 710 citations were identified through our initial search. After removal of duplicates and screening of titles and abstracts, 82 articles were retained for full text review, of which 67 were excluded. Main reasons for exclusion were that the studies did not contain data on MNM (18), did not report any relevant data (16), did not apply the WHO MNM criteria (12) or were duplicate publications from the same database (8) (Fig. 1).

Methodological quality of the remaining 15 studies is shown in Table 2. Matching in design and appropriate handling of missing data were not applied in all studies. Overall, four studies were rated as strong [29–32], three as moderately-strong [33–35] and eight as moderately-weak [11, 36–42].

**Table 2:** Methodological Quality of included cross sectional studies

Author, year	Sample							Measurement			Statistical Analysis				Total Points	Score	Quality
	Probabilistic sample used	Representative	Sample size appropriate for power	Sample drawn > 1 site	Matching design	Statistically adjusted	Response rate > 50%	DV measurement	DV reliability	DV validity	Appropriate tests used	*p* values reported	CI values reported	Missing data managed appropriately			
Ayele, 2014	0	1	1	0	NA	0	1	1	1	1	1	0	0	NA	7/13	0.54	Mod weak
Litorp,2014	0	2	1	1	NA	1	1	1	1	1	1	0	1	NA	11/13	0.85	Strong
Nelissen, 2013	0	1	1	0	NA	0	1	1	1	1	1	1	0	NA	8/13	0.62	Mod weak
Oladapo, 2015	1	2	1	1	NA	0	1	1	1	1	1	1	0	NA	11/13	0.85	Strong
Rulisa, 2015	0	1	1	0	NA	0	1	1	1	1	1	0	0	NA	7/13	0.54	Mod weak
Soma-Pillay, 2015	0	2	1	1	NA	0	1	1	1	1	1	0	0	NA	9/13	0.69	Mod strong
Tunçalp, 2013	0	1	1	0	NA	0	1	1	1	1	1	0	0	NA	7/13	0.54	Mod weak
Herklots, 2017	0	1	1	0	NA	0	1	1	1	1	1	1	0	NA	8/13	0.62	Mod weak
Kiruja, 2017	0	1	1	0	NA	0	1	1	1	1	1	1	0	NA	8/13	0.62	Mod weak
Kalisa, 2016	0	1	1	0	NA	0	1	1	1	1	1	0	1	NA	8/13	0.62	Mod weak
Nakimuli, 2016	0	2	1	1	NA	0	1	1	1	1	1	1	1	NA	11/13	0.85	Strong
Liyew, 2017	0	2	1	1	NA	0	1	1	1	1	1	0	1	NA	10/13	0.77	Mod Strong
Sayinzoga, 2017	0	2	1	1	NA	0	1	1	1	1	1	1	1	NA	11/13	0.85	Mod Strong
Mbachu, 2017	0	1	1	0	NA	0	1	1	1	1	1	1	0	NA	8/13	0.62	Mod weak
Peprah, 2015	1	2	1	1	NA	0	1	1	1	1	1	1	1	NA	12/13	0.92	Strong

Total Points = 13 total points possible; DV = Dependent Variable; CI = Confidence Interval; Weak (≤0.50), Moderate-weak (0.51 to 0.65), Moderate-Strong (0.66 to 0.79), or Strong (≥0.80)

All studies were cross sectional in design, although sometimes reported as being prospective or retrospective cohort studies. The median MNM ratio was 24.2 per 1000 live births and ranged from 4.4 in a population-based study from South Africa to 198 per 1000 live births in a private rural referral hospital in Nigeria. For each maternal death, 6.2 MNM cases were reported ranging from 1.3 in Zanzibar to 15.4 in Rwanda (IQR 2.6–6.8). Mortality index ranged from 6% in Rwanda to 43% in Zanzibar with a median of 14 (IQR 12.9–27.7). The maternal mortality ratio ranged from 71 in South Africa to 2875 per 100,000 live births in Rwanda (Table 3).

**Table 3:** Characteristics of included studies (*n* = 15)

Author, year	Country	Study setting	# Sample	# MNM	# MD	MNMr	MMR	MNM:MD	MI(%)
Ayele, 2014	Ethiopia	District hospital	8509	206	23	24.2	270	9	10
Herklots, 2017	Zanzibar	Tertiary	4125	37	28	9*	679	1.3	43
Kalisa, 2016	Rwanda	Rural referral	3994	86	13	21.5	326	6.6	13.1
Kiruja, 2017	Somaliland	Referral	1355	120	18	88.6	1328	6.7	13
Litorp, 2014	Tanzania	tertiary & regional	13,121	467	77	35.6	587	6.1	13.9
Liyew, 2017	Ethiopia	Tertiary and secondary	29,697	238	–	8	–	–	–
Mbachu, 2017	Nigeria	Private referral	262	52	5	198.5	1908	10.4	8.8
Nakimuli, 2016	Uganda	Tertiary and regional	25,840	695	130	26.9	503	5.3	15.8
Nelissen, 2013	Tanzania	District hospital	9136	216	32	23.6	350	6.8	12.9
Oladapo, 2015	Nigeria	tertiary (nationwide)	91,724	1451	998	15.8	1088	1.5	40.8
Peprah, 2015	Ghana	Tertiary and reg	2178	15	7	6.9	321	2.1	31.8
Rulisa, 2015	Rwanda	tertiary	1739	142	50	81.7	2875	2.8	26
Sayinzoga, 2017	Rwanda	District hospitals	5577	201	13	36	233	15.4	6
Soma-Pillay, 2015	South Africa	population based	26,614	117	19	4.4	71	6.2	14.0
Tunçalp, 2013	Ghana	Tertiary	3206	94	37	29.3	1154	2.5	28.2
Median (IQR)			5577 (2692, 19,480.5)	142 (90,227)	25.5 (14.3,46.8)	24.2 (12.4,35.8)	545 (322,1138)	6.2 (2.6,6.8)	14 (12.9,27.7)
Total			227,077	4137	1450	6.4	639	2.9	26

*IQR* Interquartile Range, *MNM* maternal near miss, *MD* maternal death, *MNMr* maternal near miss ratio, *MI* mortality index

### Applicability of the WHO MNM criteria

Eight studies discussed challenges related to using the WHO MNM criteria [11, 29, 34, 36–40]. A thorough discussion and adaptation was done in one study (Haydom near miss criteria) [37], and another study utilized these adapted criteria [34]. The Haydom criteria adapted the WHO MNM tool to a local hospital in Tanzania, Haydom Hospital. These criteria comprised of all the WHO clinical-based (*n* = 11), two out of eight laboratory-based criteria, and three out of six management-based criteria of the 2009 WHO MNM criteria. Additional criteria (admission to intensive care unit, eclampsia, sepsis/severe systemic infection, and uterine rupture), which were not part of the 2009 WHO criteria, were added and the threshold for the number of units of blood transfusion was lowered from five or more units of blood to one or more [9]. A study by Kalisa et al. reported another adaptation applied in Rwanda (the Ruhengeri Hospital criteria). In this adaptation they included all the WHO clinical criteria (n = 11), four out of eight laboratory-based criteria, and five out of six management-based criteria from the 2009 WHO MNM criteria. Additionally, admission to an intensive care unit, eclampsia, sepsis/severe systemic infection, and uterine rupture [11] were included. The remaining studies reported limitations with the use of some management-based (dialysis for acute renal failure, use of continuous vaso-active drugs) and a majority of the laboratory- based criteria (measuring pH, lactate, bilirubin, creatinine, arterial blood gas PaO2/FiO2) [29, 36, 38–40] (Table 4). Seven studies did not describe limitations with regard to the use of the WHO MNM tool [30–33, 35, 41, 42]. In general, suggested changes in near miss inclusion criteria included lowering the threshold of units of blood given for transfusion from five or more units to one or more [34, 37], four or more [32] or five or more units ordered but not transfused due to shortage [40]. Criteria that were suggested to be included were additional clinical criteria (eclampsia, uterine rupture, and sepsis or severe systemic infections) [11, 34, 37, 38]; and including admission to an intensive care unit as additional management-based criterion [11, 32, 34, 37, 38]. One study compared the WHO criteria with the Sequential Organ Failure Assessment (SOFA) score [43] and reported better utility of the WHO criteria in obstetrics [32]. SOFA is used to quantify organ dysfunction and predict prognosis of severely ill patients [44, 45]. Utility of SOFA score in women with MNM or admitted to intensive care unit was previously validated [43, 46, 47]. Details of reported limitations and suggested adaptations are summarized in Table 4.

**Table 4:** Applicability of the WHO MNM criteria and suggested adaptations

Study	Hospital type	Reported challenges or removed criteria	Adaptations made
Ayele, 2014, Ethiopia	District	Not all WHO near miss criteria were available	Reported as possible limitation only. No adaptation made or suggested
Litorp, 2014, Tanzania	Tertiary and secondary	Due to limited resources, some laboratory- and management-based criteria were not applicable (not specified)	None. But it was reported as a limitation for possible under-estimation especially at the regional hospital
Nelissen, 2013, Tanzania	District	Removed: PaO2/FiO2 < 200 mmHg; creatinine > 300 μmol/l or > 3.5 mg/dl; bilirubin > 100 μmol.l or > 6.0 mg/dl; pH < 7.1; lactate > 5 mEq/ml; loss of consciousness and ketoacids in urine; use of continuous vasoactive drugs; dialysis for acute renal failure	Included additionally eclampsia, uterine rupture, sepsis or severe systemic infection, admission to intensive care unit, reducing threshold of blood for transfusion from > 5 units to > 1 (Haydom Hospital criteria)
Rulisa, 2015, Rwanda	Tertiary	In most cases, it was impossible to meet the full WHO criteria because most of the laboratory tests used to define those events, were not performed at the hospital	Patients were include if they had severe maternal complications (not specified) or admitted to intensive care unit
Tuncalp 2013, Ghana	Tertiary	Although laboratory testing was available, often the markers were not requested on time or at all owing to the urgency of the management of these women.	No adaptation was made
Herklots 2017, Zanzibar	Tertiary	Some of the markers were not applicable to the setting especially laboratory criteria	Lowered threshold of blood transfusion from > 5 units to > 5 units transfused or ordered but not entirely given
Kalisa, 2016, Rwanda	District	Reported as not available: PaO2 /FiO2 < 200 mmHg; pH < 7.1; lactate > 5 mEq/ml; ketoacids in urine; dialysis for acute renal failure	Additionally included: eclampsia, uterine rupture, sepsis or severe systemic infection; admission to intensive care unit (Ruhengeri hospital criteria)
Sayinzoga, 2017, Rwanda	District	The WHO criteria adapted in the Haydom study was used	Used Haydom Hospital criteria

## Discussion

This review was conducted to assess the applicability of the WHO MNM criteria and related methodological challenges in sub-Saharan Africa. Eight of the 15 studies indicated presence of challenges in using the WHO MNM criteria: especially related to laboratory- and management-based criteria. Such limitations resulted in adapting and using ‘locally applicable’ criteria [9, 11, 34] by some while others are opting to use the original criteria [29].

Using the WHO MNM criteria without adaptation is preferred by those who aimed for comparing findings with other studies [29], but fear of underestimation lead others to adapt to broader criteria, hampering comparisons but possibly leading to more genuine estimation of MNM prevalence [9]. Unless standard criteria for using the WHO MNM approach in low-resource settings is developed [48], adaptations by some, while others opt not to adapt, will result in confusion on the outcome of studies and their comparability. Although adaptation to a local context is required for improving obstetric care and for producing genuine MNM estimates [6], several adaptations may further complicate MNM studies. On the other hand, one of the main reasons for using the standard WHO criteria—comparability—should consider issues of under-reporting and feasibility especially in low-income settings [8]. Therefore, there should be MNM criteria which can be uniformly applied and at the same time applicable to create a balanced trade-off [49].

Compared to studies using disease-based criteria, a high mortality index was reported in our review. This shows that the WHO criteria only picked the most severe MNM cases. For example, the studies from Zanzibar (mortality index, 43%) and Nigeria (mortality index, 40.8%) reported only 1.3 and 1.5 near misses per maternal death respectively [30, 40]. Other studies conducted during the same period using disease-specific criteria, reported much higher MNM ratios and lower mortality indices [50–52]. Although most MNM could be identified by clinical or management-based criteria [39], the WHO MNM criteria failed to identify nearly two-thirds of sustained severe acute maternal morbidity and one-third of maternal deaths even in high-income settings [53].

Some notable challenges should be considered in using the WHO MNM criteria in low-income settings: lack of blood for transfusion [54–56] and absence of infrastructure and ability to make an appropriate diagnosis [57, 58]. Transfusion of five or more units of blood, and diagnosis of MNM based on the majority of the WHO laboratory-based criteria are unlikely in most sub-Saharan Africa settings. As the ultimate goal of studying MNM is to improve quality of obstetric care [2, 6], this aim should not be compromised by the need to compare findings across studies.

To our knowledge, this is the first review to systematically synthesize the applicability of the WHO MNM criteria in sub-Saharan Africa. The use of WHO MNM criteria in sub-Saharan Africa is compounded by the need for having uniform criteria and limitations to apply some parameters related to laboratory- and management- based criteria. These considerations are affecting the use of the original criteria or making local adaptations based on the researchers’ judgement [29]—which may result in several different adaptations.

Locally adapted criteria may enable researchers to get a better estimate of the prevalence of MNM [59], but such findings could not be compared with other studies which used different criteria [12]. Similarly, using the WHO criteria is essential for having comparable findings across studies. But this may underestimate the true burden of cases as it only picks the most severe cases [53, 60].

## Conclusion

This review showed that the WHO MNM tool is not uniformly applied in sub-Saharan Africa. In eight studies challenges for using the WHO MNM tool were reported. Limited supply of blood and lack of infrastructure for performing some of the WHO laboratory-based criteria were the major challenges reported. There is a need to have a common tool for use in sub-Saharan Africa to avoid different adaptations because of the limited applicability of the WHO MNM tool.

## Additional file

Additional file 1: Search strategy. The list of data bases searched and keywords used for searching. (DOC 62 kb)

## Abbreviations

IQR: Interquartile range
MD: Maternal Death
MI: Mortality Index
MMR: Maternal Mortality Ratio
MNM: Maternal Near Miss
SRDR: Systematic Review Data Repository
WHO: World Health Organization
