# Supplementary material for: Applicability of the WHO maternal near miss tool in sub-Saharan Africa: a systematic review
Source: BMC Pregnancy Childbirth. 2019 Feb 26;19:79. doi: 10.1186/s12884-019-2225-7 (PMC6390325; doi:10.1186/s12884-019-2225-7)
Supplement: Supplementary file 1 — Search strategy. The list of data bases searched and keywords used for searching. (DOC 62 kb) [file 12884_2019_2225_MOESM1_ESM.doc]

Additional file 1: Search strategy

| **PubMed**:  (life threatening maternal morbidity[tw] OR severe obstetric[tw] OR severe maternal morbidity[tw] OR near miss[tw] OR severe acute maternal morbidity[tw]) **AND** ("africa south of the sahara"[MeSH Terms] OR sub saharan africa[tw] OR SSA[tw] OR "developing countries"[MeSH Terms] OR developing country [tw] OR developing countries[tw] OR low income countries[tw] OR low income country[tw] OR least developed countries[tw] OR least developed country[tw])  **EMBASE**:  ((('life threatening' OR severe) NEAR/5 (obstetric OR 'maternal morbidity')):ab,ti OR 'near miss':ab,ti OR 'severe acute maternal morbidity':ab,ti) **AND** ('africa south of the sahara'/exp OR 'africa south of the sahara':ab,ti OR 'sub saharan africa':ab,ti OR 'developing country'/exp OR 'developing country':ab,ti OR 'developing countries':ab,ti OR 'low income countries':ab,ti OR 'low income country':ab,ti OR 'least developed countries':ab,ti OR 'least developed country':ab,ti)  **CINAHL**:  TX (('life threatening' OR severe) AND (obstetric OR 'maternal morbidity')) OR TX “ near miss” OR TX “severe acute maternal morbidity” **AND** (MH "africa south of the sahara+") OR TX “sub saharan africa” OR TX “developing country” OR TX “developing countries” OR (MH "developing countries") OR TX “low income countries” OR TX “low income country” OR TX “least developed countries” OR TX “least developed country”  **POPLINE**:  (“maternal near miss” or “severe maternal morbidity” or “severe acute maternal morbidity” or “severe obstetric” or “life threatening maternal” **AND** “africa sub saharan”)  **African Journals Online (AJOL)**:  ‘‘near miss’’ OR “severe maternal morbidity” OR “severe acute maternal morbidity” OR “severe obstetric” OR “life threatening maternal”  **Google scholar**  “severe maternal morbidity” AND “sub Saharan Africa” OR “maternal near miss” AND “sub Saharan Africa” OR “severe acute maternal morbidity” AND “sub Saharan Africa” |
| --- |
